# Acute Acalculous Cholecystitis Associated with Epstein–Barr Infection: A Case Report and Review of the Literature

**DOI:** 10.1155/2020/9029601

**Published:** 2020-01-23

**Authors:** Konstantinos Ntelis, Dimosthenis Mazarakis, Apostolos Sapountzis, Dimitra Zissi, Sophia Sparangi, Nikoletta Xidia, Dimitrios Velissaris

**Affiliations:** ^1^Department of Rheumatology, General Hospital of Patras “St. Andrews”, Patras, Greece; ^2^Department of Internal Medicine, General Hospital of Kalamata, Kalamata, Greece; ^3^Department of Gastroenterology, General Hospital of Kalamata, Kalamata, Greece; ^4^Department of Microbiology, General Hospital of Sparta, Sparta, Greece; ^5^Department of Internal Medicine, University Hospital of Patras, Rion, Greece

## Abstract

The most common cause of acute cholecystitis (ACC) is cholelithiasis. Acute acalculous cholecystitis (AAC) is well documented in the literature related with critical illness, but viral causes such as cytomegalovirus (CMV) and Epstein–Barr virus (EBV) have also been reported. We present a rare manifestation of EBV infection, reporting a case of a 15-year-old female suffering from acute acalulous cholecystitis, and we review the relevant literature. Clinicians should be aware of this rare complication of EBV infection and properly exclude it in young patients with cholecystitis.

## 1. Introduction

Infectious mononucleosis (IM) is a common disease affecting mainly adolescents and young adults, usually causing fever, sore throat, fatigue, lymphadenopthy, and splenomegaly. The etiology of the disease is a widely disseminated DNA virus, the Epstein–Barr virus (EBV), a member of the herpes virus family. The genome of the virus consists of approximately 85 genes located on a double-strand DNA helix of 177.000 pair of bases [1]. It is estimated that 90% of the global population has been exposed to the virus, but the vast majority of the individuals remain asymptomatic during the infection. When symptoms are present, they are usually mild and the syndrome is self-limited.

EBV has a lymphotropic behavior, primarily affecting B cells and secondarily epithelial cells. In vitro, the expression of the viral genome inside the affected lymphocytes gives them the potential to become immortal [2]. Additionally, the virus has the ability of latent infection of a subset of memory B cells; therefore, it can remain lifelong hidden in lymphoid cells, initiating an interplay between the virus and immune system response [3]. The dysregulation of this interaction between the virus and the immune response gives a rationale for the association of EBV infection with certain autoimmune conditions [4, 5]. In addition, the expression of the latent genes of the virus in the infected cells is considered the key factor for its oncogenic activity [2].

As mentioned above, EBV infection has an asymptomatic course or it is accompanied by symptoms of IM. The main laboratory finding in IM is the extreme lymphocytosis, due to the effect of the virus in the specific CD8 T cells. The infection usually resolves without sequelae, but in a minority of cases, severe complications such as airway obstruction, meningoencephalitis, hemolytic anemia, thrombocytopenia, and splenic rupture can develop. Hepatic involvement in IM is very common, but cholecystitis is extremely rare. Herein, we report a case of acalculous cholecystitis due to EBV infection and we review the relevant literature.

## 2. Case Report

A 15-year-old female was hospitalized in the Internal Medicine Department of the General Hospital of Kalamata, Western Greece. Initially, she presented to the emergency department complaining of a 4-day history of low-grade fever up to 38.5°C, sore throat, mild cough, sharp abdominal pain, mainly in the epigastric area, and nausea. Physical examination revealed sensitivity in palpation in both right and left upper abdomen quadrants, normal bowel sounds, and no abdominal distention. Additionally, bilateral cervical and axillary lymphadenopathy, tonsillar enlargement with exudates, and a palpable spleen were noticed. Liver size was within normal range, and there was no jaundiced colouring of the sclera. Laboratory findings on admission and during hospitalization are shown in Table 1.

Abdominal ultrasound revealed thickening of the gallbladder wall to 20 mm and pericholecystic edema. Distention of the gallbladder was not evident. The spleen was enlarged to 16 cm, and the liver size was within upper normal limits. No stones or dilatation of the biliary tract were reported. Based on these findings, the diagnosis of acute acalculous cholecystitis was made and the patient was admitted to the Internal Medicine Department for further investigation and treatment. The patient was treated with the administration of intravenous fluids and antibiotics (cefoxitin 1 gr tds and clindamycin 600 mg qds). The patient manifested intense nausea and a couple of vomiting episodes on day 2 and 3 of hospitalization. Her cardiovascular and respiratory function remained stable. At the same time, liver enzymes remained elevated equally to the levels of admission. In the follow-up ultrasound, no remarkable changes were reported. Blood and urine cultures were negative. Serological tests, both heterophile antibody test and IgM antibodies against EBV capsid antigen, confirmed the diagnosis of infectious mononucleosis. Serological tests for hepatitis A, B, C, CMV, HIV, and Toxoplasma Gondi were negative. Seven days after admission, the patient was on a good clinical status, fever was resolved, the appetite had improved, and the abdominal pain had also disappeared. A new ultrasound examination on day 6 of admission revealed a total remission of the previous abnormal findings. The patient was discharged on day 8 in a good clinical status. Reassessment 2 weeks later in the Outpatients clinic of the Hospital revealed no clinical or laboratory abnormalities.

## 3. Literature Review

### 3.1. Methods

To identify relevant publications of interest, we conducted a PubMed search on December 2018 using the terms “Epstein–Barr, infectious mononucleosis, EBV, and cholecystitis.” For the purposes of this review, we limited the search to “Humans” and considered only manuscripts referring to adults. Articles in languages besides English were excluded. We also reviewed the bibliographies of all identified manuscripts to identify additional relevant publications. For the purposes of this review, we included all types of publications, including case reports, case series, and review articles, regardless of publication date.

### 3.2. Results

From the electronic search, we identified 89 articles published in English language. Fifty-three articles reported pediatric cases (patients below 14 years of age) or were irrelevant to our subject and were removed from our search results. Check for duplicates removed 13 more articles, and finally 23 case reports were left and were included in our review. The manual search returned six more cases. Overall, we collected 29 articles describing 30 cases of AAC due to EBV infection in adult patients. The literature search results are summarized in Table 2.

Clinical presentation, laboratory and imaging findings, and complications and outcomes of EBV-related AAC have been reviewed extensively in the past in a couple of reviews [23, 30]. Abdominal pain remains the most common symptom. Sore throat and pharyngitis, lympadenopathy, abdominal tenderness, and Murphy's sign are also common findings. Elevation of ALT, AST, ALP, and bilirubin in various degrees is also reported. The most common imaging finding is an increase in gallbladder wall thickness. From our search, we identified 20 cases in which antibiotics were administered. All but two patients were treated conservatively. One immunosuppressed male patient underwent cholecystectomy, and his course was complicated by acute cholestatic hepatitis, cold agglutinin-associated hemolytic anemia, and pneumonia. Histology findings were not reported [10]. The second patient who underwent surgery was a 25-years-old immunocompromised female [33]. The postoperative course was uncomplicated. The pathology report of the removed gallbladder showed AAC. All patients had a full recovery the following weeks after hospitalization.

Twenty-nine from the 31 adult patients described in the literature were females. Only two patients underwent surgery. In the first patient for whom surgery was performed, operation probably aggravated the patient's course. The patient received high doses of corticosteroids and had a good outcome after 19 days of hospitalization [10]. Another patient who also received corticosteroids improved rapidly [29]. In the second patient who underwent surgery, operation improved her symptoms [33].

Acalculous cholecystitis represents 5–10% of all cases of cholecystitis [34]. The etiology of acalculous cholecystitis is diverse. Main causes are critical illness, sepsis, trauma, surgery, severe burns, parenteral nutrition, prolonged fasting, cancer, infections (both viral and bacterial), and autoimmune disorders, such as systemic lupus erythematosus and systemic vasculitis [35, 36]. Viral agents and especially EBV are not considered common causes of acute acalculous cholecystitis, but sporadic cases have been previously described in the literature.

The diagnosis of acute acalculous cholecystitis, especially in critically ill patients, remains a clinical challenge. Clinical and laboratory findings combined with imaging findings will finally lead to diagnosis. Fever and right upper quadrant abdominal pain are the major clinical manifestations. Leucocytosis and abnormal liver tests (AST, ALT, ALP, *γ*-GT, and bilirubin) are also present, but not specific for cholecystitis. Ultrasound criteria include gallbladder wall thickness, pericholecystic fluid (halo)/subserosal edema, intramural gashydrops, gallbladder distention, and the presence of echogenic bile. Ultrasound remains the most useful utility for the diagnosis of ACC, but in uncertain cases, computed tomography and cholescintigraphy using ^99m^Tc are reasonable imaging alternatives to establish the diagnosis [35].

Due to the diverse etiology of AAC, different theories about its pathophysiology have arisen. When ACC is the result of critical illness or surgery, the related mechanisms are hypovolemia, shock, ischemia, and secondary bacterial infection from intestinal Gram-negative bacteria [37]. In cases that AAC follows prolonged fasting or parenteral nutrition, bile stasis is considered the key involved factor [37]. Bile stasis results to increased intraluminal gallbladder pressure and secondary decreased blood perfusion, ischemia, and inflammation. In sepsis, the release of proinflammatory mediators to the systemic circulation, the dissemination of the infectious agent to the bile and the concurrent immunodeficiency might explain the reports of AAC [38]. In cases of autoimmune diseases, ischemia due to visceral vasculitis is the implicating causative agent [39]. Endothelial dysfunction and vasculitis are also implicated in cases that AAC is the result of uncommon infections, such as leptospirosis and scrub typhus [40, 41]. It is obvious that in a patient with AAC more than one among the aforementioned mechanisms can be present, depending on the patient's underlying disease.

In cases of acalculous cholecystitis due to EBV infection, the pathophysiology still remains obscure. Direct invasion of the gallbladder, as EBV can infect epithelial cells, has been proposed, but this theory has not been clearly proved in the few cases that surgery was performed [10, 23, 33, 42]. Fretzayas et al. based on scintigraphic data proposed that cholecystitis due to EBV infection may not represent a true cholecystitis, but rather a billiary dyskenisia [43]. This theory is not fully accepted, as in many cases, there was just a mild elevation of ALP and bilirubin, not compatible with severe biliary pathology [23]. The most intriguing observation reviewing the cases of EBV-related cholecystitis is that, in the vast majority of the cases, the patient's gender is female. The most adequate explanation is that production of eicosanoids, which play a role in the pathogenesis of gallbladder disease, seems to be related to gender and estrogen levels [23, 44].

## 4. Discussion

The fact that female gender predisposes for certain autoimmune conditions has provided us the motive to further explore the hypothesis that EBV cholecystitis may represent some kind of autoimmune manifestation of IM, taking in mind the lymphotropic behavior of the virus and the virus interaction with the human immune system. Reviewing the literature, we identified one case of EBV cholecystitis where inflammation of the gallbladder has been accompanied by a skin rash, due to dermal vasculitis. The vasculitis was documented with a biopsy, showing lymphocytic infiltrates surrounding the walls of small vessels in the dermis accompanied by plumping of endothelial cells [29]. Another report of EBV cholecystitis accompanied by renal dysfunction with massive proteinuria referred to a 2-year-old patient. The authors underline the key role of the host immune response to EBV in the extent and degree of clinical features of the primary EBV infection [45]. The hypothesis that EBV cholecystitis is an abnormal immune response to primary EBV infection is also supported by the favorable results that glucorticoids had on the course of acalculous EBV cholecystitis in a couple of complicated cases [10, 29].

An adequate number of vasculitis reports caused by EBV infection are identified in the literature [46–51]. It is interesting that some of these cases represent localized forms of vasculitis affecting only a single organ. Barrett et al. published in 2015 the histological findings of seven patients with Epstein–Barr Virus Vulvar Ulceration (Lipschütz Disease). All but one specimen exhibited a lymphocytic arteritis (lymphocytes infiltrating and disrupting the artery wall). A diffuse and/or angiocentric predominately lymphocytic infiltrate was found in all cases [49]. Ulcers finally resolved in a short period, usually without the need of medical intervention.

Viral causes of vasculitis besides EBV are also described in the literature. Based on the severity of the clinical presentation, some of the patients with virus-triggered vasculitis received only supportive treatment, while others received antivirals and glucorticoids. Patients' outcomes were generally positive, suggesting that a benign self-limited course is the natural history of virus-associated vasculitis. This could be another clue that comes to an agreement with the benign course of the patients with EBV cholecystitis, such as described in our patient. In addition, single organ gallbladder vasculitis is a well-described form of localised vasculitis [52]. Viruses such as HBV and HCV have been identified as causative agents in some of these cases. It would be reasonable to speculate that an analogous mechanism could explain gallbladder involvement in cases of EBV-associated acalculous cholecystitis.

We report herein the case of an acalculous cholecystitis in a female adolescent, and we support the hypothesis that EBV-related vasculitis may be the underlying pathogenetic mechanism. Therefore, treatment with glucorticoids could be a useful therapeutic option. The lack of histopathology specimens makes our hypothesis difficult to be proven. Perhaps further studies in the future will elucidate the exact mechanism, which causes this rare manifestation of EBV infection. We hope that our paper will be a motivation for our colleagues to retrospectively review unpublished or published cases of EBV-related AAC that have undergone surgery and investigate the validity of our hypothesis.

## 5. Conclusion

Acalculous cholecystitis is a well-recognized complication of EBV primary infection, and clinicians should be aware of this rare clinical entity. The female gender seems to strongly predispose for this complication. In the vast majority of the cases, inflammation subsides without the need of surgical intervention and vasculitis may be the major underlying mechanism. Further larger case series and histologic confirmation are warranted.

## Figures and Tables

**Table 1:** Laboratory test results during the course of hospitalization.

Day of hospitalization	1st	2nd	3rd	4th	5th	8th (discharge day)
White blood cells (/mm^3^)	10.18	10.01	8.64	8.1	5.82	5.11
Neut (%)	16.9	13.8	20.8	14.8	17.7	24.4
Lymph (%)	66.7	70.8	65.0	75.2	69.1	64.2
PLT (10^3^/mm^3^)	120	120	140	160	189	177
Hct (%)	33.3	31.6	33.9	33.2	33.1	33.8
AST (IU/L)	106	93	95		78	
ALT (IU/L)	217	172	144		108	
ALP (IU/L)	421	406	326		348	
*γ*-GT (IU/L)	177	171	151		176	
TBIL (mg/dL)	0.84	0.54				
DBIL (mg/dL)	0.32	0.20				

Neut: neutrophils, lymph: lymphocytes, PLT: platelets, AST: aspartate aminotransferase, ALT: alanine aminotransferase, ALP: alkaline phosphatase, *γ*-GT: gamma glutamyltransferase, TBIL: total bilirubin, DBIL: direct bilirubin.

**Table 2:** Clinical and demographic characteristics of the present and previous cases of EBV-related cholecystitis.

Authors, year	Age (years), sex	Country	Treatment	Surgery
1. Yoshie et al. [6], 2004	15 female	Japan	Supportive	No
2. Koch et al. [7], 2007	53 female	The Netherlands	Supportive	No
3. Iaria et al. [8], 2008	18 female	Italy	Antibiotics	No
4. Pelliccia et al. [9], 2008	14 female	Italy	Supportive	No
5. Hagel et al. [10], 2009	21 female	Germany	Antibiotics/steroids	Yes
6. Cholongitas et al. [11], 2009	19 female	Greece	Supportive	No
7. Yang et al. [12], 2010	20 female	Korea	Antibiotics	No
8. Chalupa et al. [13], 2009	22 female	Czech Republic	Antibiotics	No
9. Arya et al. [14], 2010	16 female	USA	Antibiotics	No
10. Nagdev and Ward [15], 2011	18 female	USA	Antibiotics	No
11. Beltrame et al. [16], 2012	29 female	Italy	Antibiotics	No
12. Dylewski [17], 2012	22 female	Canada	Antibiotics	No
13. Carrascosa et al. [18], 2012	22 female	Spain	Supportive	No
14. Strehle et al. [19], 2014	14 female	UK	Antibiotics	No
15. Celik et al. [20], 2014	48 female	Turkey	Antibiotics	No
16. Gagneux-Brunon et al. [21], 2014	18 female	France	Antibiotics	No
17. Gagneux-Brunon et al. [21], 2014	20 female	France	Antibiotics	No
18. Pawlowska-Kamieniak et al. [22], 2015	17 female	Poland	Antibiotics	No
19. Agergaard and Larsen [23], 2015	34 female	Denmark	Antibiotics	No
20. Alkhoury et al. [24], 2015	15 female	USA	Supportive	No
21. Branco et al. [25], 2015	16 female	Portugal	Antibiotics	No
22. Majdalani et al. [26], 2016	16 female	Lebanon	Antibiotics	No
23. Ono et al. [27], 2016	33 female	Japan	Antibiotics	No
24. Koufakis and Gabranis [28], 2016	21 male	Greece	Supportive	No
25. Sheybani et al. [29], 2016	13 female	Iran	Steroids	No
26. Yesilbagz et al. [30], 2017	30 female	Turkey	Antibiotics	No
28. Cameron et al. [31], 2018	18 female	Canada	Supportive	No
29. Hohn et al. [32], 2018	24 male	Germany	Supportive	No
30. Rezkallah et al. [33], 2018	25 female	USA	Supportive	Yes
31. Present case	15 female	Greece	Antibiotics	No
